# Experiences of cancer survivors with chemotherapy-induced peripheral neuropathy in the Netherlands: symptoms, daily limitations, involvement of healthcare professionals, and social support

**DOI:** 10.1007/s11764-023-01402-4

**Published:** 2023-05-24

**Authors:** Daniëlle L. van de Graaf, Vivian Engelen, Aize de Boer, Gerard Vreugdenhil, Tom Smeets, Marije L. van der Lee, Hester R. Trompetter, Floortje Mols

**Affiliations:** 1 CoRPS - Center of Research on Psychological disorders and Somatic diseases, Department of Medical and Clinical Psychology, Tilburg University, PO Box 90153, 5000 LE Tilburg, The Netherlands; 2 Department of Research, Netherlands Comprehensive Cancer Organisation (IKNL), Utrecht, The Netherlands; 3 Dutch Federation of Cancer Patient Organisations, Utrecht, The Netherlands; 4 Department of Internal Medicine, Máxima Medical Centre, Veldhoven, Eindhoven, The Netherlands; 5 Centre for Psycho-Oncology, Scientific Research Department, Helen Dowling Institute, Bilthoven, The Netherlands

**Keywords:** Chemotherapy-induced peripheral neuropathy, Symptoms, Daily limitations, Healthcare professionals, Social support, Self-reported

## Abstract

**Purpose:**

A significant proportion of cancer patients suffer from chemotherapy-induced peripheral neuropathy (CIPN). This descriptive study aimed to examine patients’ experience of CIPN symptoms, daily limitations, involvement of healthcare professionals, and social support.

**Methods:**

Cross-sectional data have been collected in the Netherlands via a national online questionnaire comprising closed items only (February 2021).

**Results:**

Out of 3752 respondents, 1975 received chemotherapy only (i.e., without targeted therapy) and were therefore included. The majority (71.2%) reported symptoms in both hands and feet (e.g., tingling and loss of sensation or diminished sensation). Participants reported most limitations in household chores, social activities, hobbies, sports, walking, and sleeping and least in family/(taking care of) children, cycling, driving, self-care, eating and drinking, and sexuality and intimacy. Many patients indicated that their healthcare professionals informed them about the possibility of CIPN development before treatment (58.4%), and they paid attention to CIPN during and after treatment (53.1%). However, many patients (43%) reported a lack of information on what to do when CIPN develops. Few participants (22%) visited their general practitioner (GP) for CIPN. In general, patients’ social environments sometimes to always showed empathy to patients.

**Conclusions:**

Symptoms of CIPN are frequently reported and can result in various daily limitations. Support from professionals and peers is crucial in managing CIPN, which is sometimes lacking. Appropriate guidance and support should be provided to patients to decrease the impact of CIPN on daily life. Future research should investigate differences in chemotherapeutic agents and the resulting symptoms and consequences.

## Introduction

In the Netherlands, 123,672 new patients were diagnosed with cancer in 2021 [1]. The 5-year survival rate currently is 66% and increases by about 1% each year due to improved diagnostics and treatment [2]. The long-term consequences of cancer and its treatment become more prevalent [3], which means that after completion of treatment, patients often face several physical and psychosocial limitations in their daily lives [4–6]. Although symptoms usually improve or disappear over time, in some patients, the symptoms remain, leaving them with chronic symptoms [7].

A common long-term consequence of cancer treatment is chemotherapy-induced peripheral neuropathy (CIPN), which is caused by chemotherapeutic agents like taxanes, platinum compounds, and vinca alkaloids [8, 9]. Peripheral neuropathy is defined by the National Cancer Institute as: “a nerve problem that causes pain, numbness, tingling, swelling, or muscle weakness in different parts of the body”[10], which can be experienced as both painful and nonpainful [11]. The sensations patients experience can vary greatly depending on patient characteristics and perceptions, as well as chemotherapy type and cumulative dose [11]. One month after completion of chemotherapy, almost 80% of cancer survivors experience CIPN, which decreases to 30% after 6 months or longer [3, 8, 12–15], showing that it is still present in a significant group of patients until late after chemotherapy.

CIPN symptoms can be difficult to deal with and bring significant limitations to patients’ daily lives [16]. Even though patients are often cured of cancer, they might still feel ill due to CIPN as it continuously reminds them of being treated for a life-threatening disease [17]. Earlier research has shown that CIPN strongly interferes with daily life activities, such as walking, hobbies, and relationships [11]. Patients’ identities can change due to CIPN, as they have to make changes in their roles because certain activities can no longer be performed (e.g., performing a job or playing an instrument) [17]. Furthermore, several studies showed that CIPN symptoms can negatively affect physical, social, and emotional areas of life [11, 16, 18, 19]. This shows that CIPN can be a highly relevant limiting side effect of chemotherapy with major consequences on daily life that affects more than just physical functioning. However, to our knowledge, no earlier study examined the specific daily limitations of a broad patient group (e.g., multiple tumor types) with a large sample size.

It is crucial to be aware of the symptoms and daily consequences these patients experience as well as the attempts they make to control or reduce these symptoms since this knowledge enables healthcare professionals to adequately support patients in their needs [11]. Healthcare professionals should assess and address the symptoms of CIPN, weigh the impact on the daily lives of their patients and, subsequently, provide appropriate support to try to preserve their quality of life (QoL) [11]. A Dutch study examined reasons of colorectal cancer patients to visit their GP during the first 5 years of follow-up and concluded that chemotherapy-related symptoms, among which was CIPN, was one of the most frequent reasons [20]. However, to the best of our knowledge, no study has assessed how patients perceive support from healthcare professionals regarding CIPN.

In addition to support from healthcare professionals, support from friends, family, and significant others (i.e., social support) is important [21]. Social support can alleviate several disease aspects, such as coping with cancer and stress [22–27], anxiety, depression, and QoL [28]. A recent study among breast cancer patients examined the effect of perceived social support on chemotherapy-related symptoms, including CIPN symptoms [29]. Results showed that these symptoms were identified as less severe when patients reported medium to high perceived social support, compared to those with low perceived social support. However, the relevant CIPN symptoms examined in the study were pain and numbness, whereas CIPN involves a much broader spectrum of symptoms. It is important to examine the social support experienced by cancer survivors. In this study, social support is referred to as the degree of empathy shown by the social environment.

This study aimed to report the experiences of cancer survivors who suffer or had suffered from self-reported CIPN in the Netherlands regarding (1) CIPN symptoms, (2) daily limitations, (3) involvement of healthcare professionals, and (4) social support.

## Methods

### Study design

A cross-sectional exploratory national online questionnaire study was performed among adult cancer survivors with CIPN in the Netherlands. The questionnaire was initiated and developed by a patient advocate and a researcher from the Dutch Federation of Cancer Patients Organizations (NFK), which is the Dutch umbrella organization that represents 19 cancer patient organizations. A researcher of the PROFILES Registry with scientific expertise in CIPN was also involved in the development of the questionnaire [30]. Furthermore, four patient advocates of two patient organizations (the Dutch breast cancer patient organization (*Borstkankervereniging Nederland*) and the Dutch gynaecologic cancer patient support group (*Stichting Olijf*) were involved, three of whom experienced CIPN themselves. These people participated in a workgroup. The workgroup met three times to discuss the content of the questionnaire. In between, the workgroup provided (digital) feedback on draft versions of the questionnaire.

### Data collection

The questionnaire was distributed between February 1 and 15, 2021 via www.doneerjeervaring.nl, social media channels and the *Doneer Je Ervaring* (Donate Your Experience) panel. Additionally, patient organizations have spread invitations for the questionnaire among their members and sponsors via email. Finally, partner organizations like the Dutch Cancer Society and Kanker.nl (Dutch web platform with tailored medical information and peer-support targeted at cancer survivors and relatives [31]) have spread the invitation.

### Participants

Patients could participate if they currently suffered from CIPN or had suffered from CIPN in the past. Participants were informed about privacy regulations of the NFK, in accordance with the General Data Protection Regulation (EU). The Medical Research Involving Human Subjects Act (WMO) did not apply since the study did not include an intervention wherefore ethical approval by the Medical Ethical Review Board was not needed. By completing the questionnaire, patients gave implied consent. Participation was completely online and anonymous.

### Questionnaire

The questionnaire started with four closed-ended questions regarding demographics. The remaining 29 closed-ended questions included the following topics: CIPN sensations, daily limitations, attention to CIPN by healthcare professionals, and social support. In questioning the daily limitations, cycling was included as a separate category since cycling is one of the most widely used means of transportation in the Netherlands, making it a daily activity for many people and not just a sports activity.

### Statistical analyses

No minimum sample size was calculated prior to the study since this study was explorative in nature. Descriptive statistics were reported. Absolute numbers and percentages were provided for nominal variables. Means and standard deviations were provided for continuous variables. For both items related to satisfaction with the supervision of healthcare professionals, the numerical scores 1 to 10 were recoded to the categorical scores insufficient (1–5), satisfactory to good (6–8), and excellent (9–10). IBM SPSS Statistics version 28 was used for all analyses.

## Results

In total, 3752 participants filled in the questionnaire. Participants were included if they had received “chemotherapy only” as treatment. Excluded were participants that received “targeted therapy,” “targeted therapy combined with chemotherapy,” or neither of those. In total, 1975 participants who suffered from of had suffered from CIPN remained and were included in this study. Sociodemographic characteristics of participants are shown in Table 1. Participants had a mean age of 58.8 (*SD* = 11.3), and 76.1% were female. The most prevalent tumor types were breast (42.8%), blood/lymph (24.2%), colorectal (8.9%), and gynecological cancer (5.8%). In 41% of patients, cancer had been diagnosed more than 5 years ago, followed by 2–5 years ago (34.7%) and less than 2 years ago (23.6%).

**Table 1:** Sociodemographic characteristics of participants

Characteristics	*n*	%	*M*	*SD*
Gender				
Male	469	23.7		
Female	1506	76.1		
Other	5	.3		
Age			58.8	11.3
Cancer type				
Breast	848	42.8		
Blood/lymph	480	24.2		
Colorectal	177	8.9		
Gynecological	115	5.8		
Other	90	4.5		
Lung	56	2.8		
Prostate	44	2.2		
Bladder/kidney	37	1.9		
Pancreas	35	1.8		
Testicle	35	1.8		
Stomach/esophageal	31	1.6		
Head/neck	10	.5		
Sarcoma	9	.5		
Brain	9	.5		
Melanoma	4	.2		
Time since diagnosis				
< 2 years	468	23.6		
2–5 years	687	34.7		
> 5 years	825	41.7		

### CIPN symptoms

More than half of the participants reported to suffer from CIPN for more than 2 years at the time of the questionnaire (55.1%) (Table 2). In 71.2% of the participants, CIPN was present in both foot/feet and hand(s). The most prevalent symptoms in hands were tingling (59.6%) and loss of sensation or diminished sensation (47.7%). Most participants attempted to reduce or control these symptoms by applying self-management strategies (69.9%).

**Table 2:** Experienced symptoms of CIPN

	*Number*	***%***
Duration of symptoms		
A few weeks	82	4.1
A few months	195	9.8
About half a year	168	8.5
About 1 year	198	10.0
About 2 years	164	8.3
More than 2 years	1090	55.1
Don’t know (anymore)	83	4.2
Location of symptoms		
Foot/feet	423	21.4
Hand(s)	147	7.4
Foot/feet and hand(s)	1410	71.2
Type of symptoms in feet		
Tingling	1327	67.0
Loss of sensation or diminished sensation	1360	68.7
Changed sensation	1043	52.7
Pain	758	38.3
Pain from touch	457	23.1
Pain from temperature changes	669	33.8
Burning or stabbing pain	664	33.5
Balance disorders	592	29.9
Muscle weakness or reduced strength	552	27.9
Muscle cramp	716	36.2
Thinning of muscles	215	10.9
Type of symptoms in hands		
Tingling	1180	59.6
Loss of sensation or diminished sensation	944	47.7
Changed sensation	724	36.6
Pain	486	24.5
Pain from touch	293	14.8
Pain from temperature changes	587	29.6
Burning or stabbing pain	318	16.1
Balance disorders	93	4.7
Muscle weakness or reduced strength	653	33.0
Muscle cramp	334	16.9
Muscle loss	137	6.9
Attempted to reduce or cope with symptoms		
Yes	1384	69.9
No	532	26.9
I don’t know/not applicable	64	3.2

### Daily limitations

**Fig. 1:**
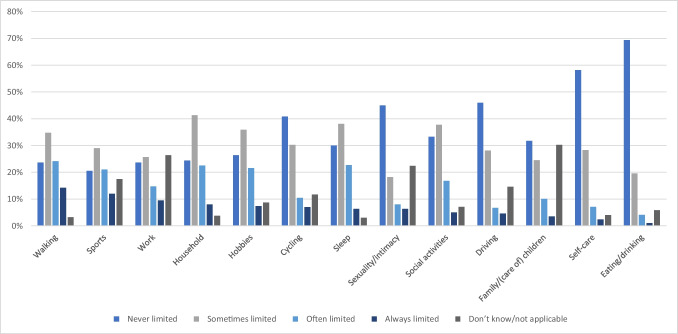
Daily limitations due to CIPN

Figure 1 shows the reported daily limitations due to CIPN. In several types of daily activities, most patients report never experiencing limitations, namely, eating and drinking (69.4%), self-care (58.2%), driving (46%), sexuality and intimacy (45%), cycling (40.8%), and family/(taking care of) children (31.7%). In the remaining categories, limitations were more common. Being “sometimes limited” was most reported by participants for household chores (41.3%), sleep (38.1%), social activities (37.8%), hobbies (35.9%), walking (34.8%), sports (29.0%), and work (25.7%).

### Involvement of healthcare professionals

Table 3 shows an overview of hospital healthcare professionals’ and GPs’ attention to CIPN. Regarding hospital healthcare professionals, more than half of the participants (58.4%) reported being informed before treatment about the possibility of the development of CIPN. Also, most participants (72.7%) reported that attention was given to CIPN symptoms during or after treatment. However, many (43%) reported not being informed about what to do when CIPN develops. Satisfaction with attention to CIPN was rated as insufficient by almost a quarter of patients (23%). However, many patients rated it as satisfactory to good (50.3%), or as excellent (26.7%).

**Table 3:** Attention to CIPN by healthcare professionals

Hospital healthcare professional	*N*	*%*
Before treatment: informed about possibility of CIPN^a^ development		
Yes	1157	58.4
No	523	26.4
Don’t know (anymore)/not applicable	300	15.2
During or after treatment: attention to CIPN^a^		
Yes	1439	72.7
No	541	27.3
During or after treatment: informed about what to do when CIPN^a^ develops		
Yes	716	36.2
No	852	43.0
Don’t know (anymore)/not applicable	412	20.8
Satisfaction with attention to CIPN^ab^		
Insufficient (1–5)	417	23.0
Satisfactory-good (6–8)	914	50.3
Excellent (9–10)	485	26.7
General practitioner	*N*	***%***
During or after treatment: visited general practitioner for CIPN^a^		
Yes	436	22.0
No	1481	74.8
Don’t know (anymore)/not applicable	63	3.2
Satisfaction with attention to CIPN^ab^		
Insufficient (1–5)	89	21.2
Satisfactory-good (6–8)	224	53.3
Excellent (9–10)	107	25.5

^a^*CIPN* chemotherapy-induced peripheral neuropathy

^b^Due to none-obligatory nature of item, valid percentages were reported

Only a small proportion of the participants (22%) reported having visited their GP for CIPN. The majority rated the GP attention to CIPN as satisfactory to good (53.5%), followed by excellent (25.5%). Few indicated this as insufficient (21.2%).

### Social support

**Fig. 2:**
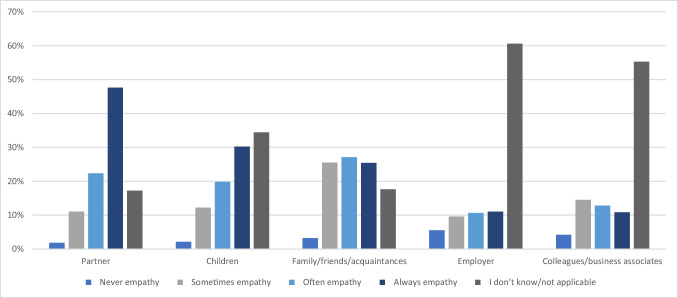
Social support and empathy

Participants indicated that partners showed empathy regarding CIPN always (47.6%), often (22.3%), and sometimes (11%) respectively (Fig. 2). Only a small minority reported that their partner never showed empathy (1.8%). This also applied to children (30.2%, 19.8%, 12.2%, 2.1% respectively). In the case of family, friends, and acquaintances, participants reported that empathy was shown often (27.1%), sometimes (25.5%), and always (25.4%), respectively, followed by a small minority of participants who reported that empathy was never shown (3.2%). Most participants indicated “I don’t know/not applicable” for social support regarding colleagues and business associates (55.3%) as well as for employers (60.6%). However, the remaining participants indicated that empathy was shown never (4.2%), sometimes (14.5%), often (12.8%), and always (10.8%) by colleagues and business associates. In the case of employers, participants reported that empathy was shown never (5.5%), sometimes (9.6%), often (10.6%), and always (11%).

## Discussion

The aim of this study was to explore patients’ experience of CIPN symptoms, daily limitations, involvement of healthcare professionals, and social support. Although symptoms are mostly experienced in both hands and feet and are often non-painful (e.g., tingling and loss of sensation or diminished sensation), a significant part of participants reports painful symptoms. These symptoms can result in a variety of daily limitations, in which most patients are able to perform activities of daily life (ADL) but seem to experience problems mainly in their roles and social activities. Support and empathy are not always experienced by patients. This mainly applies to support and empathy from healthcare professionals, friends and acquaintances, and work-related peers.

This study has shown that even though most patients with CIPN experience non-painful symptoms, a significant proportion of people (also) experience painful symptoms. Earlier research into colorectal cancer survivors has shown that QoL and physical, role, cognitive, and social functioning are worse in patients with painful CIPN compared with patients with non-painful CIPN [18]. However, the effect of painful versus non-painful CIPN on the daily limitations people experience was not examined in our study. For this reason, and since painful versus non-painful CIPN may involve different impairments and coping mechanisms, research on the difference in daily limitations and appropriate psychosocial interventions between painful and non-painful CIPN is needed.

Results of this study have shown that most patients experience limitations in daily life, which vary in how often people experience them. For example, daily activities such as eating and drinking, self-care, cycling, intimacy and sexuality, and driving are not limiting for a reasonable group of patients. Therefore, some patients seem able to perform the ADL themselves. However, there are also many ADL in which many patients often experience limitations, such as walking, sleeping, and household chores. Previous research has indeed shown that patients with CIPN might become limited in various aspects of functioning (i.e., physical, social, emotional, role, and cognitive), which in turn deteriorates their QoL [13, 14, 16, 18, 32]. Patients should therefore receive support from healthcare professionals and peers. This may increase the (sense of control over their) ability to perform daily activities independently, contributing to patient empowerment, which can improve QoL [33–35]. However, results should be interpreted with caution as no comparison in daily limitations between patients with CIPN and the general population has been made. Therefore, it is not clear what the CIPN-specific limitations are, as the general population may also experience limitations given the relatively high average age in this sample.

Furthermore, this study showed that patients indicate they were not informed about what to do when CIPN develops. It is not known whether information has not been provided to patients, or whether patients were informed but had different priorities in the process of facing a life-threatening disease. This means that for many patients, a search for symptom self-management begins when CIPN symptoms arise. Research has shown that patients often lack knowledge and self-management skills to properly manage their cancer-related pain [36]. Several studies have shown that psychoeducation for cancer-related pain can positively influence patients’ knowledge and ability to self-manage their symptoms [37–41]. Applying symptom self-management must be supported by healthcare professionals [42], which starts with informing patients appropriately and providing advice, starting before treatment. Furthermore, options and wishes in dose reduction of chemotherapy should be considered during treatment to possibly limit development or worsening of CIPN [8, 43–47]. However, no evidence-based treatment recommendations can currently be provided as there are no effective treatments for non-painful CIPN [45, 46].

In addition, patients should also be supported in self-management by their social environment [42]. Our study showed that most patients often feel empathy by their social environment mainly by partners, family, and friends, in which the degree of empathy varies. Earlier research has shown that such support improves CIPN and coping with cancer [27, 29]. However, our study showed that empathy by colleagues and employers is often lacking. Work-related social support includes both organizational support from employers (e.g., job security, flexible working hours, and sick pay) and interpersonal support from colleagues (e.g., empathy and positive attitudes) [48–51]. Since work-related social support is crucial in achieving work-related goals and returning to work after cancer [48–51], more attention must be paid to social support from colleagues and employers. However, in this study, colleague and employer support was not applicable for most participants since many patients are probably retired given the high average age of the sample. Therefore, these results should be interpreted cautiously, and further research on work-related social support is needed.

Furthermore, even though our research shows that many patients often or always feel empathy with respect to CIPN by family, friends, and acquaintances, there is still a significant group of patients who never or only sometimes feel empathy is shown. Earlier research has found that 52% of breast cancer patients experienced to be sometimes avoided or contact is feared by friends and family [52]. Interestingly, this study also examined the perspective of healthy people, which showed that 61% of them would or might avoid people with cancer. Reasons of relatives for not providing social support to cancer patients appear to be diverse and can include, for example, the perception of one’s own inability to provide support, as well as not wanting to burden the cancer patient emotionally [53]. However, it has been shown that patients wish to receive social support, and they experience increased QoL when they receive helpful social support [54]. However, social support appears to diminish significantly within 1 year after diagnosis [54], which could possibly explain the lack of empathy regarding CIPN experienced by some of the patients in our study, since CIPN can be present for a long time after treatment [3, 8, 12–15]. Another explanation may be that relatives often do not know what CIPN entails and do not understand the symptoms [55]. Because of the variety of symptoms, it can be difficult to understand and explain CIPN to family, friends, and acquaintances. Healthcare professionals should provide appropriate information to patients about CIPN even before treatment has started, so that patients can properly explain their symptoms when they arise [55], thus creating more openness and awareness about CIPN.

### Strengths and limitations

This study has several strengths. First, this was a nationwide study with a high number of participants. Second, a strength of this study was the variety in the time since diagnosis and the type of cancer of the participants. The distribution of participants in less or more than 2 years after diagnosis is nearly equal. Thus, any coasting effect of CIPN (i.e., unexpected decrease or increase in CIPN symptoms in the weeks or months after the last dose of chemotherapy [56]) in this sample can be considered less relevant.

Some limitations also need to be discussed. First, the questionnaire was probably mainly filled in by patients that are connected to patient organizations, which might not be representative of all cancer patients. Second, it also appears to be an unrepresentative sample in terms of distribution in tumor types. This applies, for example, to lymphoma, which involves a much lower percentage of patients in the Netherlands than in this sample. It also applies to breast cancer, which explains the high number of women in this sample. As women and men have different coping strategies in general [57] and relating to cancer specifically [58], which may also affect the daily limitations they face, this may have affected the results of daily limitations. Third, the questionnaire was only available in Dutch, which prevented non-Dutch-speaking residents from completing the questionnaire. Fourth, a non-validated questionnaire was used. Fifth, only physical, role, and social aspects of functioning were considered in examining daily limitations. Future research should also look at emotional and cognitive functioning. Sixth, fatigue was not taken into account in the assessment of daily limitations, while fatigue is one of the most common side effects of cancer treatment [59]. Seventh, the sample is very heterogeneous and no analyses regarding differences between chemotherapeutic agents could be made since no data regarding chemotherapeutic agents was collected. Future research should examine comparisons between chemotherapeutic agents and associated symptom and consequences.

## Conclusion

This exploratory study showed that patients with CIPN suffer from various symptoms which may result in daily limitations. The prevalence of these limitations differs, and, as a result, the extent to which patients are able to perform ADL also varies. The degree of attention to, and satisfaction with, this attention from healthcare professionals varies, which also applies to the level of empathy from the social environment. Appropriate guidance from healthcare professionals, starting before treatment, and support from the social environment is crucial in enabling patients to feel empowered in their daily lives despite CIPN.

## Data Availability

Data are available from and with permission from Dutch Federation of Cancer Patient Organizations (NFK).
